# Surface qualities have little effect on vection strength

**DOI:** 10.3389/fpsyg.2014.00610

**Published:** 2014-06-25

**Authors:** Masaki Ogawa, Chihiro Hiramatsu, Takeharu Seno

**Affiliations:** ^1^Department of Human Science, Faculty of Design, Kyushu UniversityFukuoka, Japan; ^2^Institute for Advanced Study, Kyushu UniversityFukuoka, Japan; ^3^Research Center for Applied Perceptual Science, Kyushu UniversityFukuoka, Japan

**Keywords:** vection, self-motion, surface quality, material category, spatial frequency, subjective impression

## Abstract

We investigated the effects of different surface qualities of materials on vection strength. Previous studies have extensively examined the stimulus parameters for effective vection induction. However, the effects of surface qualities on vection induction have not been studied at all despite their importance in realistic perception of a scene. As a first step toward understanding the effects of surface qualities on vection, we investigated surface qualities derived from light-reflecting properties of nine material categories commonly encountered in daily life: bark, ceramic, fabric, fur, glass, leather, metal, stone and wood. To relate vection strength with low-level visual features and with subjective impression of materials, we analyzed spatial frequency and participants' ratings of adjective pairs that describe impressions of material categories. Although the nine material categories were perceived differently, there was no main effect of material condition on vection strength. However, multiple regression analyses revealed that vection was partially explained by both spatial frequency and principal components extracted from the subjective impression. These results indicate that although the effect of surface qualities of materials on vection is small, both low-level image-based and perceptual-level processing of surface qualities may influence vection^1^.

## Introduction

Exposure to optical motion that simulates the retinal optical flow generated by self-movement commonly causes the perception of subjective movement of one's own body. If the same retinal optical flow occurs without self-movement, the illusory percept of self-motion known as “vection” may result (Fischer and Kornmüller, 1930). For example, when people observe a stationary train beginning to move in some direction, they are likely to perceive that they are moving in the opposite direction. This phenomenon, known as the train illusion, provides a good illustration of vection.

Analyzing the factors that affect vection strength is the central issue of vection studies because understanding these factors is critical for explaining the ecological function of vection. In previous studies, effective stimulus properties for vection induction have been examined extensively. For example, the visual field and its effect on vection induction have been under investigation since the first experimental study of vection by Brandt et al. (1973). Many studies have consistently reported that wider and larger visual fields induce stronger vection (e.g., Brandt et al., 1973; Held et al., 1975; Lestienne et al., 1977). Also, several reports have indicated that the peripheral visual field is more effective than the central field for vection induction (Brandt et al., 1973; Held et al., 1975; Johansson, 1977; Dichgans and Brandt, 1978).

Stimulus depth also plays an important role in vection. Many studies have reported that as stimulus distance increases, so does vection strength (Delorme and Martin, 1986; Ohmi and Howard, 1988; Howard and Heckmann, 1989; Ito and Shibata, 2005). Ito and Shibata (2005) presented two optic flow stimuli (contraction and expansion) at different depth planes simultaneously. Under these conditions, the farther plane dominated the direction of vection; i.e., if the expansion appeared farther away than the contraction, vection was induced in the forward direction (and vice versa). These studies indicated that vection is more heavily influenced by motion that occurs farther from the observer.

The addition of color to stimuli also seems to increase vection strength. Bonato and Bubka (2006) reported that a grating consisting of six different colors induced stronger vection than did a black and white grating. Similarly, Bubka and Bonato (2010) reported stronger vection for a natural scene with color than for the same scene without color. Furthermore, Nakamura et al. (2010) examined the effect of dynamic color changes on the induction of vection. The study used an optic flow with dots whose color alternated either randomly or coherently at 1 Hz between red and gray. Vection magnitude was much weaker in the coherent color change condition than in the random condition. Finally, Seno et al. (2010) reported that the color red induces weaker vection than does green.

Spatial frequency also influences vection. Palmisano and Gillam (1998) investigated the interaction of spatial frequency and the eccentricity of the stimulus. They found that moving patterns of low (0.11 cycle/degree) vs. high (0.2 cycle/degree) spatial frequency enhanced peripherally-mediated vection.

It is also known that vection strength is modulated by implicit meanings of the moving components of a scene (e.g., Seno and Fukuda, 2011; Ogawa and Seno, in press). For example, small dots with a simple round shape that are falling induce the illusory percept of upward self-motion, i.e., vection, whereas small petal shapes falling can induce the subjective experience of being under cherry trees as their blooms drop. This cognitive bias might inhibit vection induction (Ogawa and Seno, in press).

In sum, stimulus attributes for effective and ineffective vection induction were studied extensively in the 1970 s and 1980 s, and studies on the contributions of color to vection stimuli were added in the 2000 s. More recently, the cognitive aspects of vection have been examined (e.g., Schulte-Pelkum et al., 2004; Riecke et al., 2006; Seno and Fukuda, 2011). However, there is another set of attributes of visual stimuli that has not been studied at all, namely surface qualities.

Every object in the world has surface qualities; indeed, we are able to visually perceive these objects by analyzing the reflection of light from their surfaces. Therefore, surface qualities are essential visual attributes for grasping the real world. In the past, surface qualities were thought to be difficult to examine scientifically. However, more recently many researchers have begun to study them, thanks to the development of computer graphic technologies that enable us to create stimulus images with realistic surface qualities.

Surface qualities include various visual features such as color, texture, and light reflection/transmission properties (e.g., glossiness and translucency) and considerable research has investigated these features (Motoyoshi et al., 2007; Anderson and Kim, 2009; Doerschner et al., 2010; Kim and Anderson, 2010; Motoyoshi, 2010; Marlow et al., 2011, 2012; Kim et al., 2012). For example, Motoyoshi et al. (2007) reported that simple statistics such as skew of the visual stimulus are important for perceived gloss. The series of articles by Kim et al., however, reported that perceived gloss cannot be determined solely by simple statistics, but depends on the structure of images due to the interaction of light with different surface properties (Anderson and Kim, 2009; Kim and Anderson, 2010; Marlow et al., 2011, 2012; Kim et al., 2012). Although surface qualities have been gaining importance as attributes of visual stimuli, there is no empirical information currently available about how surface qualities may influence vection. Our study focuses on the categorical perception of surface qualities and spatial frequency. We included spatial frequency because it has been implicated as a simultaneous modulator of both vection strength and perception of surface qualities.

Surface qualities are a viable subject of investigation for vection modulation not only because they have not previously been examined but also because these qualities are related to a realistic representation of the world, which might influence the perception of self-motion. In recent vection studies, it has been reported that more natural stimuli can induce stronger vection. For example, Riecke et al. (2006) showed that a natural scene can induce stronger vection than a scrambled natural scene. The same study demonstrated that an inverted natural scene was less effective than an upright natural scene. Finally, Schulte-Pelkum et al. (2004) reported that naturalistic stimuli induced stronger vection than did non-naturalistic stimuli. If we wish to consider the degree to which visual stimuli appear realistic and natural, we should focus on the image properties associated with surface qualities. However in previous vection studies, stimuli usually have been dots or gratings whose image properties were not naturalistic.

In this study, we examine whether surface qualities of materials influence the induction and strength of vection. We hypothesize that the different surface qualities would induce different strengths of vection. Because the process of categorizing materials is ecologically important, in this initial step toward understanding the effects of surface qualities on vection, we focused on surface qualities of materials that we encounter in daily life. Sharan (2009) showed that humans are extremely good at identifying material categories such as metal and stone, even in rapid presentations. She also reported that the contribution of low-level image features such as spatial frequency in the process of categorizing materials is small. A brain imaging study that examined how patterns of neural activity reflect the low-level image processing or high-level perceptual representation of material categories demonstrated that early visual areas are responsible for low-level image processing of materials and higher ventral visual areas are related to perceptual categories of materials (Hiramatsu et al., 2011). These findings suggest that categorizing materials involves more than low-level processing of images.

Previous studies have shown that bottom-up factors such as spatial frequency of stimuli and top-down cognitive analysis of a scene both modulate vection. Arguably, perception of surface and material properties also involves both bottom-up and top-down processes in the brain, and those processes would likely influence induction and strength of vection differently. It is interesting to examine how low-level visual features such as spatial frequency and high-level perceptual impression of material categories are related to induction and strength of vection. Therefore, we focused on spatial frequency and subjective impressions of the surface qualities of materials as a low-level and a high-level factor that might influence vection. We thought that differential effects on vection of stimulus spatial frequency will provide evidence of bottom-up processes and differential effects of impression of materials on vection will provide evidence of top-down processes. These are specific and testable, and also strengthen the motivation for the study (i.e., to determine whether vection is a process mediated by both bottom-up and top-down factors).

## Materials and methods

### Materials

#### Ethics statement

The study was pre-approved by the Ethics Committee of Kyushu University.

#### Apparatus

Stimuli were generated and controlled by a computer (ALIENWARE-M18x, Dell, Austin, TX) and presented on a plasma display (3D Viera 65-inch, Panasonic, Japan, with 1920 × 1080 pixel resolution at a 60-Hz refresh rate). The experiment was conducted in a dark chamber.

### Methods

#### Participants

Fifteen adult volunteers participated. Participants were graduate and undergraduate students and the two authors (aged between 21 and 35 years; 8 males and 7 females). All were of sound physical and mental health, with normal color vision and eyesight, and no history of any of the following conditions: ear pain or headaches when boarding aircraft, vestibular system diseases, cardio-respiratory diseases, moderate balance disorders, dizziness, or altitude sickness. Except for the two authors, no participants were aware of the purpose of the experiment.

#### Visual stimuli

**Figure 1:**
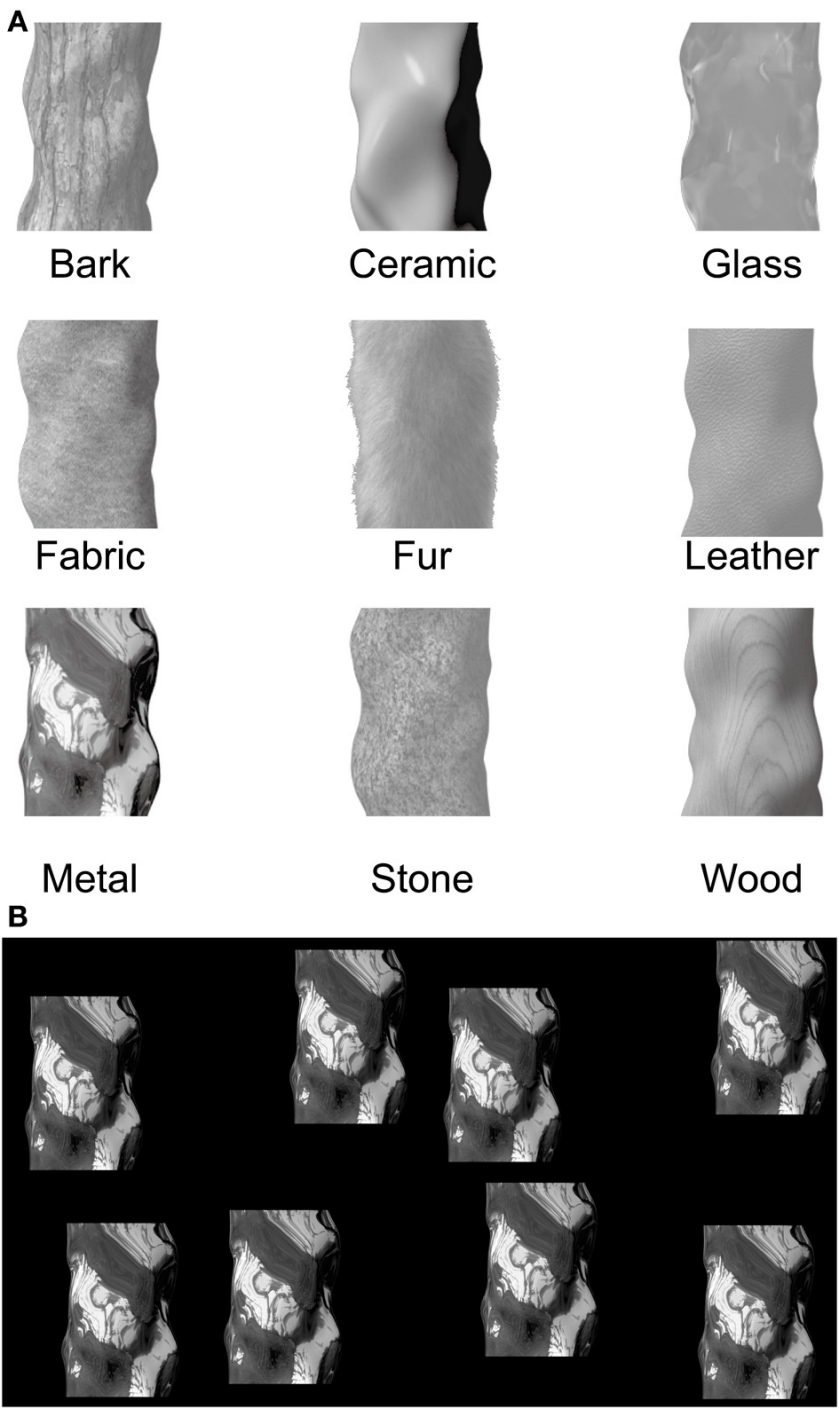
**Visual stimuli used in this study. (A)** The images of nine surface qualities. Each image corresponds to a material category we encounter in our daily life. **(B)** The vection stimulus consisted of eight objects moving rightward.

Virtual 3D images created by LightWave 3D computer graphic software (NewTek, San Antonio, TX) were used in this study (Figure 1A). Each image was of an object with a slightly different abstract shape and a surface quality from one of nine material categories: bark, ceramic, fabric, fur, glass, leather, metal, stone, or wood. The nine images had previously been used in an fMRI study (Hiramatsu et al., 2011) and were converted into gray scale and resized to 400 × 400 pixels (30 × 30 cm, ca. 28 × 28 degree at a 60-cm viewing distance) for the current study. The mean luminance was equated for each of the nine images at 6.4 cd/m^2^. The viewing screen measured 100.2 × 71 degree. There was no fixation point, and participants' heads were not fixed by a chinrest.

We should note here the reason for employing these nine surface qualities. Sharan (2009) conducted an annotation study to determine the most common materials in everyday experience, using photographs of daily scenes. She found that metal, fabric, wood, glass, plastic and stone are surface categories frequently observed in our daily environment. Considering that surface qualities naturally vary within each category, she constructed a material database with exemplars from the following categories: metal, fabric, wood, glass, plastic, stone, paper, water and leather (Sharan, 2009). On the basis of this study, Hiramatsu et al. (2011) used computer graphics to create a set of stimuli that comprised nine material categories, with shape as a controlled variable. The categories included ceramic, bark and fur instead of plastic, paper and water. Plastic was eliminated because it can appear similar to other materials such as ceramic, glass and Japanese lacquerware (*urushi-nuri*); thus it was difficult to create typical plastic stimuli. In the case of paper, it was difficult to create various types of paper surfaces with computer graphics, and the thinness of paper shapes was not compatible with the shapes of other categories. Water was also not compatible with rendering stable objects. Therefore, Hiramatsu et al. (2011) included ceramic and bark because both have considerable opportunity for variations without sacrificing the typical perception of the materials. Fur and skin were considered for the ninth category because both are considered biologically important. In the end, fur was chosen owing to the relative ease of creating realistic stimuli, especially when rendered on the abstract shapes.

Following Hiramatsu et al. (2011), we chose to use isolated objects (components) rather than global motion components, e.g., the moving wall. Hiramatsu and colleagues showed that the expected subjective impressions of surface quality could be rendered on these isolated objects. Anderson and Kim (2009) reported that shape (three-dimensionality) can be a critical factor for perceived surface qualities, e.g., gloss. Therefore, we purposely chose not to alter the shape (3D quality) of the components used by Hiramatsu et al. (2011).

Because examining the effects on vection of the surface qualities of material categories was the main focus of this study, we did not control the image statistics that inevitably differ across categories. We controlled only the mean luminance of stimuli and did not control other image statistics such as contrast and skew, nor did we change the highlight position of images, because manipulations of those parameters would have greatly impaired naturalistic perception of the material categories. Rather, we focused on how categorically perceived materials influence vection and how low-level visual features and high-level impressions might mediate vection.

On each trial, one of the images was selected randomly for presentation. Eight identical instances of this image (hereafter, “objects”) appeared together on a uniformly black background (0 cd/m^2^, 1920 × 1080 pixels). The eight objects did not overlap in space. The objects moved rightward with a constant velocity (33.3 degree/sec) (Figure 1B). These stimuli were intended to induce leftward vection.

#### Procedure

Participants were asked to press a button whenever they perceived leftward self-motion, and to keep the button depressed for the duration of the perceived self-motion. We recorded the latency and duration of vection. After each trial, participants rated the subjective vection strength using a 100-point rating scale where 0 represented no vection and 100 represented very strong vection. These procedures have been used in our previous studies (Seno, 2013a,b). The duration of the stimulus was fixed at 30 seconds. Each condition was repeated four times; thus there were 36 trials in total. The order of conditions was randomized.

After finishing a vection session, participants made subjective ratings of each surface quality. Each quality was instantiated in a single, large, stationary image that was presented in the center of the screen (about 29.1 × 29.1 degree with little difference between the nine surface qualities). We employed a rating method similar to the SD method of Osgood et al. (1957). We used 12 visual and non-visual adjective pairs: matte–glossy, opaque–transparent, simple–complex, regular–irregular, fancy–modest, smooth–rough, dry–wet, cold–warm, soft–hard, light–heavy, elastic–inelastic, and natural–artificial. Previous studies (Holliins et al., 1993; Rao and Lohse, 1996; Picard et al., 2003; Hiramatsu et al., 2011) have demonstrated that these adjective pairs are well suited for characterizing the nine material categories. We also used two additional adjective pairs, alive–dead and static–dynamic, which seem suitable for evaluating whether a surface quality looks animate or inanimate. All adjectives were presented in Japanese. The participants were asked to rate each image using a seven-level scale for each adjective pair.

### Analysis

To examine whether there was a main effect of material condition, and whether specific surface qualities inhibited or enhanced vection strength, we conducted a one-way repeated measures analysis of variance (within-subjects).

We also evaluated whether there were relationships between vection and subjective impressions or low-level image features. For measures of subjective impressions, we applied a principal component analysis (PCA) on the mean ratings of the 14 adjective pairs for each category across participants to extract a reduced set of principal components (PCs) that would account for most of the variance in the ratings.

We chose spatial frequency as the low-level image feature to analyzed because there is evidence (Palmisano and Gillam, 1998) that it may be related to vection. Specifically, we analyzed the prevalence of low, middle, and high spatial frequency, using the Steerable Pyramid, linear multi-scale, multi-orientation image decomposition filter (Portilla and Simoncelli, 2000). We first decomposed the textural portion (i.e., not including the outline between object and background) of each image (192 × 192 pixels) into three scales (low, middle and high) and four orientations and then obtained the mean magnitude of spatial frequency across the four orientations for each scale. Although the cutoff values for high and low spatial frequencies are not obvious in this analysis, high spatial frequencies correspond to as those between approximately 1.8 and 3.6 cycle/degree, and low spatial frequencies correspond to as those between approximately 0.4 and 0.9 cycle/degree. We calculated the proportion of magnitude of high frequency compared with that of low frequency and used it as a measure of prevalence of high spatial frequency. This analysis was conducted by the MATLAB texture analysis program provided by the following website: http://www.cns.nyu.edu/~lcv/texture/.

Using the measures derived from these analyses, we conducted multiple regression analyses to examine how low-level image-based and high-level perceptual-based measures of material categories were related to vection. In the analysis, vection measures (latency, duration and magnitude) for each material condition were dependent variables, proportion of high spatial frequency to low spatial frequency (SF-ratio) and scores of PCs for each material condition were independent variables. First, we explored the best model with the smallest Akaike's information criterion value by including the all independent variables in the analysis. If the best model included more than two independent variables, then we added an interaction (moderation) term to the best model by multiplying the standardized values of two independent variables. If the model including the interaction term yielded a higher *R*-squared value than that of the best model without the interaction term, we defined the model with the interaction term as the best model. This kind of moderation analysis would yield significant results for interaction terms if there were interactions between spatial frequency and subjective impression of material categories.

To describe possible correlations between subjective impression and vection measures, we conducted the Pearson correlation analysis between each vection measure and individual ratings of each adjective pair for each material condition. Because there were possibilities of Type I errors due to the number of correlation analyses, we only used *r*-value correlation coefficients as measures of effect size to describe the strength of each correlation. We also conducted correlation analyses between the mean vection measure and the mean ratings across participants in each material condition for each adjective pair.

Statistical analyses were conducted by R (version 3.0.2) or MATLAB R2010b (MathWorks, MA).

## Results

**Figure 2:**
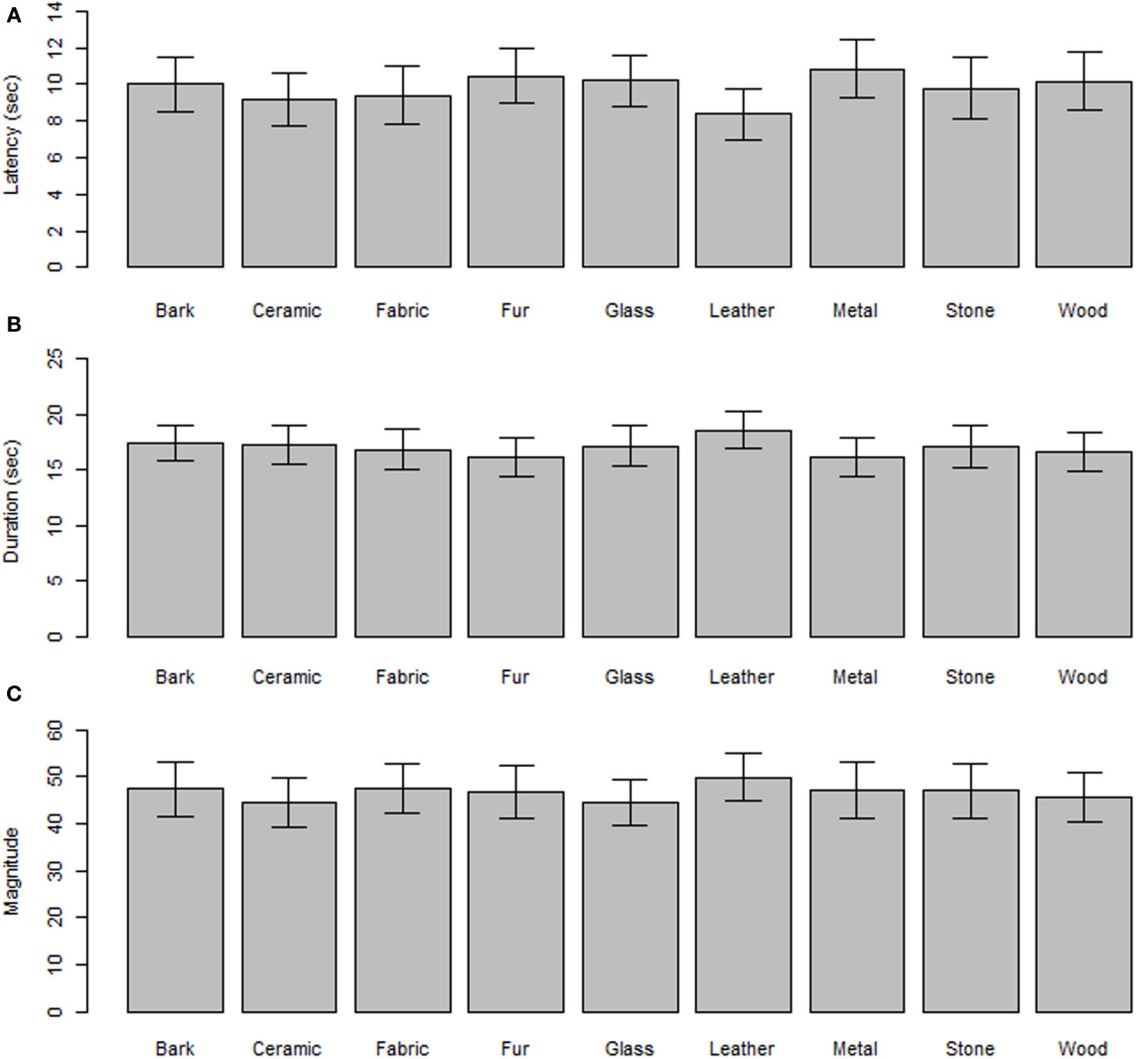
**Three measures of vection as a function of nine material category conditions. (A)** The latency of vection induction. **(B)** The duration of vection. **(C)** The estimated magnitude of vection.

Figure 2 shows the results for the three vection measures: latency (Figure 2A), duration (Figure 2B) and magnitude (Figure 2C) in the nine material conditions. There was a trend toward greater vection strength in the leather condition and lesser strength in the glass and metal conditions. However, the main effect of material was not significant for any of the three vection measures [Latency, *F*_(8, 112)_ = 1.33, *p* = 0.23; Duration, *F*_(8, 112)_ = 0.97, *p* = 0.45; Magnitude, *F*_(8, 112)_ = 0.72, *p* = 0.67].

**Figure 3:**
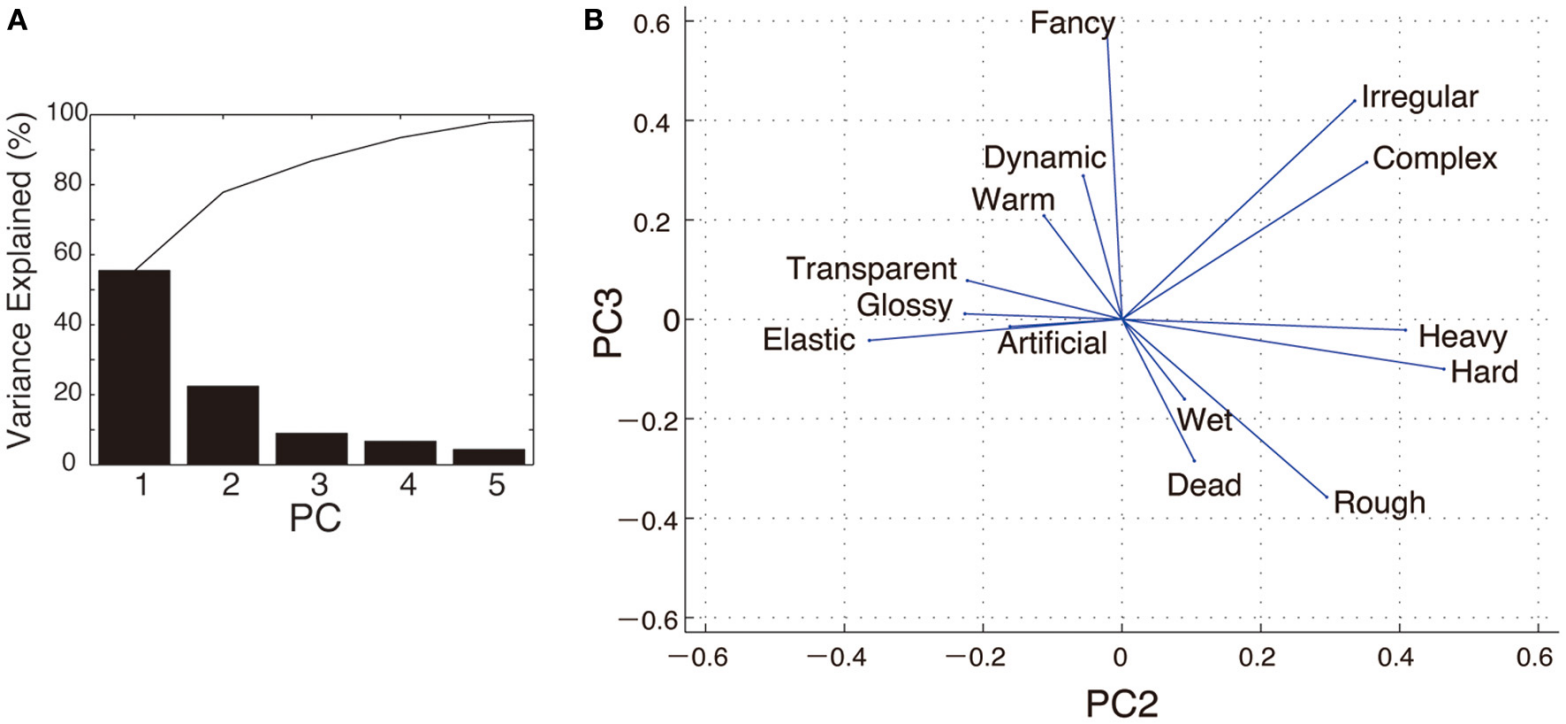
**PCA for the impressions of material images. (A)** Scree plot of the PCA. Each bar shows the variance of rating data explained by each PC (PC1–PC5). The line chart indicates the cumulative variance of the data explained by PCs from PC1 to up to PC5. **(B)** The two-dimensional plot consisting of PC2 and PC3. The coefficients of adjectives are indicated by blue lines. Note that PC1 was not shown in the space since it was not involved in the best models explaining vection measures.

In the PCA, the first three PCs explained 86% of the variance in the ratings data (Figure 3A). PC1 explained 55% of the variance, and it had high coefficients for glossy and artificial. PC2 explained 22% of the variance and had high coefficients for hard and heavy. PC3 explained 9% of the variance and had high coefficients for fancy and irregular (Figures 3A,B).

Table 1 shows the best models obtained by multiple regression analyses to describe the relationships between vection measures, SF-ratio and PCs. The best model to explain latency contained SF-ratio and PC3 (*F* = 18.2, df = 8, adjusted *R*^2^ = 0.81, *p* = 0.0029) as independent variables. The best model to explain duration only contained PC3 (*F* = 9.22, df = 8, adjusted *R*^2^ = 0.51, *p* = 0.019). The best model to explain magnitude contained PC2, SF-ratio and the interaction term, SF-ratio^*^PC2 (*F* = 37.2, df = 8, adjusted *R*^2^ = 0.93, *p* = 0.00076). We note again that PC2 had high load on hard and heavy and PC3 had high load on fancy and irregular (Figure 3B).

**Table 1:** **Relationships between vection measures, SF-ratio and PCs assessed by multiple regression analysis**.

**Dependent variable**	**Independent variable**	**Estimate**	***t*-value**	***p*-value**
Latency	SF-ratio	−8.4	−2.54	0.044
	PC3	0.36	4.14	0.0061
Duration	PC3	−0.4	−3.04	0.019
Magnitude	SF-ratio	24.2	4.32	0.0076
	PC2	0.17	2.35	0.065
	SF-ratio^*^PC2	−18.55	−4.21	0.0084

The independent variables included in the best models that explain the dependent variables are listed. Estimate indicates the estimated coefficients of each independent variable in a model.

In the Supplementary Materials, we provided the correlation coefficients and *r*-squared values between vection measures and ratings of adjective pairs.

## Discussion

In this study, we investigated how surface qualities of materials influence latency (induction), duration, and strength of vection. The nine different surface qualities were perceived as expected in the different categories, and the obtained subjective impressions were totally different across the nine. Nevertheless, there was no significant main effect of material condition on the vection measures, probably owing to large variation among individuals (Figure 2).

This main finding could be considered a negative result. However, it is important for vection research and also for practical applications, such as content development of virtual reality. There were no surface qualities that facilitated or impeded vection induction: whatever the surface quality of the vection stimulus was, vection was induced at the same strength.

It was a surprising result that although the different surface qualities can modify the meanings of the vection stimuli, those semantic modifications did not affect vection strength. We speculate that in this current study, the types of semantic modifications of vection stimuli (i.e., the categories of surface qualities) were not effective for vection modulation. However, regression analyses revealed that PCs did have some effect on vection strength; therefore, we must allow the possibility that semantic information about different surface qualities could modulate vection strength. In any case, future examination of this topic is needed, with particular attention to the relationship between the semantic meanings and surface qualities.

We included SF-ratio as a variable representing low-level visual features and PCs as variables representing subjective impressions. Although the observed effects were small, the multiple regression analyses indicated that these variables were involved in predicting vection latency and magnitude. Specifically, high SF-ratio (the ratio of higher to lower spatial frequencies) appeared to enhance vection. This result seems to be partially consistent with a previous study (Palmisano and Gillam, 1998) in which higher, rather than lower, spatial frequencies tended to enhance vection when stimuli were presented in the central visual field. However, our stimuli contained both central and peripheral motion stimulation. In addition, the ranges of spatial frequencies used in two studies were different. In this study, high and low spatial frequency corresponded to a range approximately between 1.8 and 3.6 cycle/degree and 0.4 to 0.9 cycle/degree, while in Palmisano and Gillam's study, high spatial frequency corresponded to 0.2 cycle/degree, and low spatial frequency corresponded to 0.11 cycle/degree. Therefore, the effects of stimulus spatial frequency on vection would not be similar between two studies. It is also possible that spatial frequency not only of the textural portion of objects, but of the whole visual field affected our results. Because both studies suggested a relationship between spatial frequency and vection, future research must focus on this topic to reveal the relationship more clearly. Furthermore, perceived surface qualities may differ between central and peripheral vision. Kim and Anderson (2010) showed that perceived gloss depends on high-spatial-frequency information, which cannot be perceived accurately in peripheral vision (Thibos et al., 1996). At the same time, stimuli in peripheral vision are thought to be important for vection induction (e.g., Brandt et al., 1973). Thus the relationships among stimulus eccentricity, surface qualities and vection also require more investigation.

In the multiple regression analysis, we found that PC2 and PC3 were related to vection measures. PC3 was associated with increased latency and decreased duration. This observation is consistent with the fact that latency and duration are normally negatively correlated. Considering that PC3 had high load on fancy and irregular, fanciness and irregularity of surface qualities may be related to vection induction. For magnitude, we found an interesting interaction between SF-ratio and PC2. This may indicate that the effect of SF-ratio is dependent on the subjective impression of an object's hardness or heaviness in each category since PC2 had high load on hard and heavy.

Although there was no factorial effect of material categories, our results suggest the possibility that both low-level visual features and subjective impressions of materials are related to vection. It will be interesting to see how early visual areas that process low-level visual features and higher brain areas related to perception of surface qualities contribute to vection in future imaging studies.

The moving components used in this study were more likely to be perceived as objects than as backgrounds in a scene. A material's function as figure vs. background in natural scenes may influence vection. It is also possible that vection strength depends to some extent on whether the object is perceived as dynamic or static in a scene. These aspects should be controlled to extract the effect of surface qualities independently of an object's status as figure vs. background. For example, stimuli like moving walls with different surface qualities might be useful in future experiments. In addition, although only gray scale images were used in this study, colors that occur in surface qualities of natural objects should also be considered in future studies to create more realistic observations of surface qualities on vection.

### Conflict of interest statement

The authors declare that the research was conducted in the absence of any commercial or financial relationships that could be construed as a potential conflict of interest.

## References

[B1] Anderson B. L. Kim J. (2009). Image statistics do not explain the perception of gloss and lightness. J. Vision 9, 1–17 10.1167/9.11.10 20053073

[B2] Bonato F. Bubka A. (2006). Chromaticity, spatial complexity, and self-motion perception. Perception 35, 53–64 10.1068/p5062 16491708

[B3] Brandt T. Dichgans J. Koenig E. (1973). Differential effects of central versus peripheral vision on egocentric and exocentric motion perception. Exp. Brain Res. 16, 476–491 10.1007/BF00234474 4695777

[B4] Bubka A. Bonato F. (2010). Natural visual-field features enhance vection. Perception 39, 627–635 10.1068/p6315 20677700

[B5] Delorme A. Martin C. (1986). Roles of retinal periphery and depth periphery in linear vection and visual control of standing in humans. Can. J. Psychol. 40, 176–187 10.1037/h0080091 3730954

[B6] Dichgans J. Brandt T. (1978). Visual-vestibular interaction: effect on self-motion perception and postural control, in Handbook of Sensory Physiology, eds Held R. Leibowitz H. W. Tueber H. L. (Berlin: Springer-Verlag), 755–804

[B7] Doerschner K. Boyaci H. Maloney L. T. (2010). Estimating the glossiness transfer function induced by illumination change and testing its transitivity. J. Vision 10, 1–9 10.1167/10.4.8 PMC4454136 20465328

[B8] Fischer M. H. Kornmüller A. E. (1930). Optokinetic ausgelöste Bewegungs-wahrnehmungen und optokinetinetisher Nystagmus. J. Psychol. Neurol. 41, 273–308

[B9] Held R. Dichgans J. Bauer J. (1975). Characteristics of moving visual scenes influencing spatial orientation. Vision Res. 15, 357–365 10.1016/0042-6989(75)90083-8 1136151

[B10] Hiramatsu C. Goda N. Komatsu H. (2011). Transformation from image-based to perceptual representation of materials along the human ventral visual pathway. Neuroimage 57, 482–494 10.1016/j.neuroimage.2011.04.056 21569854

[B11] Holliins M. Faldowski R. Rao S. Young F. (1993). Perceptual dimensions of tactile surface texture: a multidimensional scaling analysis. Percept. Psychophys. 54, 697–705 10.3758/BF03211795 8134240

[B12] Howard I. P. Heckmann T. (1989). Circular vection as a function of the relative sizes, distances, and positions of two competing visual displays. Perception 18, 657–665 10.1068/p180657 2602091

[B13] Ito H. Shibata I. (2005). Self-motion perception from expanding and contracting optical flows overlapped with binocular disparity. Vision Res. 45, 397–402 10.1016/j.visres.2004.11.009 15610745

[B14] Johansson G. (1977). Studies on visual perception of locomotion. Perception 6, 365–376 10.1068/p060365 917725

[B15] Kim J. Anderson B. (2010). Image statistics and the perception of surface gloss and lightness. J. Vision 10, 1–17 10.1167/10.9.3 20884601

[B16] Kim J. Marlow P. J. Anderson B. L. (2012). The dark side of gloss. Nat. Neurosci. 15, 1590–1595 10.1038/nn.3221 23001059

[B17] Lestienne F. Soechting J. Berthoz A. (1977). Postural readjustments induced by linear motion of visual scenes. Exp. Brain. Res. 28, 363–384 885185 10.1007/BF00235717

[B18] Marlow P. Kim J. Anderson B. (2011). The role of brightness and orientation congruence in the perception of surface gloss. J. Vision 11, 1–12 10.1167/11.9.16 21873616

[B19] Marlow P. Kim J. Anderson B. (2012). The perception and misperception of specular surface reflectance. Curr. Biol. 22, 1909–1913 10.1016/j.cub.2012.08.009 22959347

[B20] Motoyoshi I. (2010). Highlight–shading relationship as a cue for the perception of translucent and transparent materials. J. Vision 10, 1–11 10.1167/10.2.16 20884604

[B21] Motoyoshi I. Nishida S. Y. Sharan L. Adelson E. H. (2007). Image statistics and the perception of surface qualities. Nature 447, 206–209 10.1038/nature05724 17443193

[B22] Nakamura S. Seno T. Ito H. Sunaga S. (2010). Coherent modulation of stimulus colour can affect visually induced self-motion perception. Perception 39, 1579–1590 10.1068/p6793 21425698

[B35] Ogawa M. Seno T. (in press). Vection is modulated by the semantic meaning of stimuli and experimental instructions. Perception. 10.1068/p7639 25223105

[B23] Ohmi M. Howard I. P. (1988). Effect of stationary objects on illusory forward self-motion induced by a looming display. Perception 17, 5–11 10.1068/p170005 3205670

[B24] Osgood C. E. Suci G. J. Tannebaum P. H. (1957). The Measurement of Meaning. Chicago, IL: University of Illinois Press

[B25] Palmisano S. A. Gillam B. (1998). Stimulus eccentricity and spatial frequency interact to determine circular vection. Perception 27, 1067–1077 10.1068/p271067 10341936

[B26] Picard D. Dacremont C. Valentin D. Giboreau A. (2003). Perceptual dimensions of tactile textures. Acta Psychol. 114, 165–184 10.1016/j.actpsy.2003.08.001 14529823

[B27] Portilla J. Simoncelli E. P. (2000). A parametric texture model based on joint statistics of complex wavelet coefficients. Int. J. Comput. Vision 40, 49–70 10.1023/A:1026553619983

[B28] Rao A. R. Lohse G. L. (1996). Towards a texture naming system: identifying relevant dimensions of texture. Vision Res. 36, 1649–1669 10.1016/0042-6989(95)00202-2 8759466

[B29] Riecke B. E. Schukte-Pelkum J. Caniard F. (2006). Using the perceptually oriented approach to optimize spatial presence & ego-motion simulation, in Handbook of Presence (Hillsdale, NJ: Lawrence Erlbaum Assoc.), 49–57

[B30] Schulte-Pelkum J. Riecke B. E. Bulthoff H. H. (2004). Vibrational cues enhance believability of ego-motion simulation, in International Multisensory Research Forum (IMRF). Available online at: www.kyb.mpg.de/publication.html?publ=2766

[B31] Sharan L. (2009). The Perception of Material Qualities in Real-World Images. Unpublished Doctoral dissertation, Massachusetts Institute of Technology

[B32] Seno T. (2013a). Social inhibition of vection. Psychology 4, 619–621 10.4236/psych.2013.48088

[B33] Seno T. (2013b). Music modulates the strength of vection. Psychology 4, 566–568 10.4236/psych.2013.47081

[B34] Seno T. Fukuda H. (2011). Stimulus meanings alter illusory self-motion (vection)–experimental examination of the train illusion. Seeing Perceiving 25, 631–645 10.1163/18784763-00002394 23550369

[B36] Seno T. Sunaga S. Ito H. (2010). Red inhibits vection. Atten. Percept. Psychol. 72, 1642–1653 10.3758/APP.72.6.1642 20675807

[B37] Thibos L. N. Still D. L. Bradley A. (1996). Characterization of spatial aliasing and contrast sensitivity in peripheral vision. Vision Res. 36, 249–258 10.1016/0042-6989(95)00109-D 8594823

